# Health behaviours as predictors of the Mediterranean diet adherence: a decision tree approach

**DOI:** 10.1017/S1368980021003293

**Published:** 2022-07

**Authors:** Joana Margarida Bôto, Ana Marreiros, Patrícia Diogo, Ezequiel Pinto, Maria Palma Mateus

**Affiliations:** 1 University of Algarve – School of Health, Department of Dietetics and Nutrition, Gambelas Campus, 8005-139 Faro, Portugal; 2 Department of Biomedical Sciences and Medicine, Faro, Portugal; 3 Necton SA, Olhão, Algarve, Portugal; 4 School of Health, Department of Dietetics and Nutrition, Faro, Portugal; 5 Centre for Health Studies and Development, Faro, Portugal

**Keywords:** Mediterranean Diet, Adolescence, Health behaviours, Dietary habits, Decision trees, Machine learning

## Abstract

**Objective:**

This study aimed to identify health behaviours that determine adolescent’s adherence to the Mediterranean diet (MD) through a decision tree statistical approach.

**Design:**

Cross-sectional study, with data collected through a self-fulfilment questionnaire with five sections: (1) eating habits; (2) adherence to the MD (KIDMED index); (3) physical activity; (4) health habits and (5) socio-demographic characteristics. Anthropometric and blood pressure data were collected by a trained research team. The Automatic Chi-square Interaction Detection (CHAID) method was used to identify health behaviours that contribute to a better adherence to the MD.

**Setting:**

Eight public secondary schools, in Algarve, Portugal.

**Participants:**

Adolescents with ages between 15 and 19 years (*n* 325).

**Results:**

According to the KIDMED index, we found a low adherence to MD in 9·0 % of the participants, an intermediate adherence in 45·5 % and a high adherence in 45·5 %. Participants that regularly have breakfast, eat vegetable soup, have a second piece of fruit/d, eat fresh or cooked vegetables 1 or more times a day, eat oleaginous fruits at least 2 to 3 times a week, and practice sports and leisure physical activities outside school show higher adherence to the MD (*P* < 0*·*001).

**Conclusions:**

The daily intake of two pieces of fruit and vegetables proved to be a determinant health behaviour for high adherence to MD. Strategies to promote the intake of these foods among adolescents must be developed and implemented.

Dietary habits changed dramatically in modern times due to globalisation^(1)^. Although an abundance of food is now constantly available in developed countries, the supply of energy-dense but nutritionally poor food products has contributed to a deterioration in the quality of dietary and health habits^(2)^. As a consequence, the prevalence of several non-communicable diseases associated with an unbalanced diet, such as obesity, diabetes, CVD and hypertension, has increased^(3)^. It is estimated that non-communicable diseases account for 70 % of worldwide deaths every year^(4)^. Within the WHO European Region, approximately 89 % of deaths are attributed to non-communicable diseases, and one-third of these deaths occur pre-maturely (between the ages of 30 and 69 years)^(5)^. The onset of these diseases is currently occurring earlier in life^(6)^. Inadequate nutrition is related with a higher prevalence of obesity in children and adolescents, and childhood and adolescence obesity has become a major global public health concern, following rapid increases in many parts of the world in recent decades^(7)^.

The Mediterranean diet (MD), considered as an healthy lifestyle model that contributes to preventing and reducing obesity^(8,9)^, was recognised by UNESCO as a cultural heritage of humanity^(10)^, and the Mediterranean food pattern is a nutritional model of excellence, scientifically reported as healthy^(11–13)^ and sustainable^(14)^. Nutritionally, this food pattern is low in saturated fats, high in antioxidants, fibbers and monounsaturated fats, exhibits an adequate *n*-6/*n*-3 fatty acid balance, and is a source of phytosterols and probiotics^(15,16)^. Although several studies have linked adherence to the MD with a higher degree of protection against all-cause mortality, highlighting its protective role against the development of diseases^(17–20)^, adherence to MD has decreased as a result of the incorporation of Western country habits, following globalisation^(21)^. The decreased adherence to MD observed in Mediterranean populations is also occurring in Portugal^(22)^.

Among children and adolescents, epidemiological evidence suggests that dietary patterns in Mediterranean countries are changing rapidly, with an increase in the consumption of animal products, saturated fat, highly processed/energy-dense foods, ready-to-eat products and a decline in the intake of plant-based foods^(23–25)^. Adolescence is a key period in life, and it involves multiple physiological and psychological changes that affect nutritional needs and habits^(26)^. Adolescents have increased energy and nutrient needs, including amino acids for growth of striated muscle, as well as Ca and vitamin D to accommodate bone growth^(27,28)^. Adherence to MD, it is associated with an enhanced nutritional adequacy^(11)^ and is also recognised for preventing overweight, obesity^(29)^ and in reducing waist circumference in adolescents^(30,31)^

In Portugal, the latest data from the National Food and Physical Activity Survey, IAN-AF 2015–2016, reports that 78 % of adolescents (aged between 10 and 17 years) does not meet the WHO recommendation to consume more than 400 g/d of fruit and vegetables (equivalent to five or more servings/d). The daily consumption of more than 50 g of processed meat is observed in 6·3 % of the population (11·6 % in adolescents). Only 36 % of people between 15 and 21 years of age are considered physically active, complying with the current recommendations for the practice of ‘health-promoting physical activity’^(32)^.

Machine learning is a method focused on the development of algorithms that are particularly useful for data mining^(33)^. Data mining is a term to describe the process of analysing large databases in search of interesting and previously unknown patterns^(34)^. Decision tree methodology is a commonly used data mining method to establish classification systems based on multiple covariates or for developing prediction algorithms for a target variable. The algorithm is non-parametric and can efficiently deal with large, complex datasets without imposing a complicated parametric structure^(35)^. Decision tree structure comprises a hierarchically organised set of data groups, called nodes, which are interconnected by tree branches. The beginning of the hierarchical structure is the root of the tree (i.e. root node). It refers to the dependent variable and includes all the observed data that are to be divided into classes during the process of model development^(36)^. Machine learning uses the combination of artificial intelligence algorithm with statistical analysis for decision-making, which can be useful to support dietary behaviours understanding and improvement.

This study aims to identify which health behaviours contribute to a higher adherence to MD and provide a framework for the development of food education interventions aimed at adolescents through decision-making processes using classification trees.

## Methods

### Sampling and participants

This study focused on tenth-grade students, recruited through a randomised, multi-stage, stratified sample of schools, constructed from a sampling frame comprising all secondary schools in the region of the Algarve, Portugal. After following the procedures proposed by Fleiss *et al.* ^(37)^ for calculating minimum sample size, with 90 % power and 0·05 statistical significance, and considering official data provided by the Regional Directorate of Education regarding the number of registered tenth-grade students in the Algarve, we selected, in the first stage, a random sample of eight schools, stratified by their type – science or humanities curriculum schools and technological-professional schools; in the second stage, we randomly selected fourteen classes from science or humanities curriculum and nine classes from technological-professional schools. All students from these classes were invited to be a part of the study and a formal, written consent, was sent to their legal guardians.

The calculations for sample size suggested a minimum of 454 participants to achieve representativity. The classes randomly selected for recruitment had a total of 545 enrolled students. From the original sample of 545 students, 325 (59 6 %) were authorised by their guardian to be a part of the study and agreed to proceed to the data collection phase. Exclusion criteria were (1) pregnancy and (2) physical disability, mental illness, or other condition that affected the ability to fulfil the data collection questionnaire or the validity of anthropometric measurements. Data collection was conducted from April to June 2018. No participants were excluded on account of these criteria.

### Data assessment and variables

Data were collected through a self-fulfilment questionnaire regarding food, lifestyle and health. This tool has five sections: (1) dietary habits; (2) adherence to the MD (KIDMED index); (3) physical activity; (4) sleep and oral hygiene; and (5) socio-demographic characteristics. Except for section 2, the questions were developed specifically for this study, based on best nutritional epidemiology practices and also on national and international tools, namely the data collection questionnaires and protocols used in the Health Behaviour in School-Aged Children (HBSC)^(38)^, Childhood Obesity Surveillance Initiative (COSI)^(39)^, and the Portuguese National Food, Nutrition and Physical Activity Survey (IAN-AF)^(40)^. These tools were consistently found reliable in diverse settings and different populations.

The data collection tool was pre-tested in a homologous, non-random sample of 22 volunteers that were debriefed after fulfilling the questionnaire. This pre-test resulted in small edits and minor corrections to the questionnaire that were incorporated in the final version used in this study.

Anthropometric and blood pressure data were also collected, using procedures documented in a field manual and after specific training for all members of the team. Data included weight, height, waist and hip circumference, and systolic and diastolic blood pressure.

In all questions regarding dietary habits, physical activity, sleep and oral hygiene, participants were asked to consider the last 12 months as a frame of reference for their answers.

### Dietary habits

Dietary habits were assessed through close-ended questions, either multiple choice, ‘yes/no’ questions, Likert-type sentences for assessing agreement or questions for quantification (e.g. number of daily meals). As the goal for this assessment was to determine specific dietary habits, to be analysed per question and not by the means of a composite scale or index, questions were focused on partaking specific meals, their setting (e.g. ‘Do you usually have breakfast?’; ‘Where do you usually have breakfast?’ and ‘Who usually has breakfast with you?’) and the usual foods included in each meal, selected from a list and with the ability of participants adding their own foods. The adoption of a specific kind of diet (vegan, meat-free, Halal, etc.) and the amount of water usually ingested during the day were also assessed with multiple choice questions, with the ability of participants adding other answers. The intake of alcoholic beverages was assessed using a standard, semi-quantitative, food frequency scale.

### Adherence to the Mediterranean Diet

The adherence to the MD was assessed with the Portuguese version of the KIDMED index proposed by Mateus, MP^(41)^. The score for this index ranges from 0 to 12 and it is based on 16 ‘yes/no’ questions. Questions denoting a negative connotation with the MD are assigned a score of -1 and those positively associated with the MD are assigned a score of +1. The sum of the scores is then categorised in three levels: level 1 – high adherence to MD (≥ 8 points); level 2 – intermediate adherence to MD (4–7 points) and level 3 – low adherence to MD (≤ 3 points), as proposed by the original authors of the KIDMED index^(42,43)^.

### Physical activity, sleep and oral hygiene

Physical activity was assessed through nineteen multiple choice questions, aimed at gaining information regarding the usual means of transportation to and from school, the distance between home and school, the type of physical activity in school hours and as a form of leisure. We did not intend to quantify physical activity but instead identify participants who engage in any kind of physical activity and identify the usual forms of physical activity.

Participants were also asked to about their usual time of waking up, usual time of going to bed and usual frequency of brushing their teeth.

### Socio-demographic variables and anthropometrics

For the socio-demographic characterisation, we collected information regarding date and place of birth, gender, nationality, area of residence, as well as household composition, age, nationality, place of birth, education and profession of parents/guardians.

Anthropometric measurements were made using reference methodologies^(44)^ by trained researchers. Weight was assessed to the nearest decigram (0·1 kg) with a Seca 877® digital scale. Height was measured to the nearest millimetre (0·1 cm) with a portable Seca 217® stadiometer, with the participant’s head in the Frankfurt plane. Weight and height were measured twice, with participants in bare feet and light clothes. The waist circumference was measured up to the nearest millimetre (0·1 cm), at the midpoint between the iliac crest and the lower costal margin, using a flexible and non-deformable Teflon tape. BMI was calculated using the formula Weight/Height^2^ (kg/m^2^). The BMI was used to determine the BMI/age percentile, and participants were categorised as underweight (≤5th percentile), normal weight (>5th to ≤85th percentile), overweight (>85th percentile) and obese (≥95th percentile), according to the WHO growth standards for children and adolescents^(45)^.

### Statistical analysis

IBM-SPSS® software version 25.0 was used for statistical analysis. Data were described by absolute and relative frequencies, and mean, median (MD), standard deviation and interquartile range were computed whenever appropriate. Data were normalised by logarithmic or arcsine transformation when results were expressed as percentages. To evaluate the impact of MD adherence on each gender, while considering anthropometric data and health behaviours, we performed a one-way ANOVA multiple comparison test (Tukey, *P* < 0·05). For the variables that did not follow a normal distribution, non-parametric tests were computed, such as Kruskal–Wallis, Chi-square, Mann–Whitney and Spearman’s correlation coefficient (*P* < 0·05).

We performed a machine learning procedure as a complement to other statistical analyses in order to further study the relationship between adherence to MD and other variables. Since the variables are potentially correlated with each other, a decision tree was applied through the algorithm CHAID (Automatic Chi-square Interaction Detection)^(46)^. These tree models classify cases into groups or predict values of a dependent variable (criterion), based on values or categories of the independent variables (predictors). The criterion used was the classification of individuals having low, intermediate or high adherence to MD in relation to the following factors: dietary habits; items of KIDMED index; sleep and oral hygiene; school sports; out of school sports; leisure physical activity; daily hours of sedentary activities; socio-demographic data; and anthropometric and blood pressure measures. A maximum tree depth of three levels was generated by the algorithm with a minimum number of fifty initial (parent) nodes, thirty terminal (child) nodes, and Splitting Nodes criteria of 0·05. The algorithm was able to differentiate the groups with low, intermediate or high adherence to MD obtained by KIDMED index categories (dependent variable in node 0). All other questionnaire variables were considered independent variables. Variables were not present in the decision tree if a regression could not be generated by the algorithm; therefore, no homogenous groups were formed, and the variable was not represented in the decision tree (e.g. sleep duration). Therefore, when the algorithm can generate a new level of ramification from a node, it suggests that the new groups formed have significantly different levels of adherence to MD.

## Results

### Sample characteristics

The participants in our study (*n* 325) were between 15 and 19 years old, with a mean age of 16·4 years (sd 0·89). Fifty-three per cent (*n* 172) reported as female and 47 % (*n* 153) as male.

Regarding BMI/age percentile categories, 79·4 % (*n* 258) of the participants had normal weight and 19·7 % (*n* 64) were overweight. Girls had higher mean values for hip circumference than boys (Table 1). Boys were taller, heavier, with higher waist circumference and systolic blood pressure than girls (Table 1). Table 2 shows data on dietary habits. Boys reported a higher frequency for breakfast and after dinner meals (*P* < 0·05); girls showed a higher frequency of mid-morning meals, higher consumption of salad, vegetables and fruit at lunch and dinner, and higher median number of intermediate meals (*P* < 0·05). Bread is the most consumed food item at breakfast, mid-morning and at mid-afternoon meals, in both genders (Table 2).

**Table 1:** Anthropometric and blood pressure characteristics of the sample

	Total (*n* 325)				Female (*n* 172)				Male (*n* 153)				*P* value
	M	sd	MD	IQR	M	sd	MD	IQR	M	sd	MD	IQR	
Weight (kg)	60·3	11·13	58·6	66·3–51·8	56·7	9·92	53·8	63·2–49·7	64·3	11·1	63·3	69·4–55·8	**<0·001**
Height (m)	1·66	0·086	1·66	1·72–1·60	1·61	0·062	1·60	1·64–1·57	1·72	0·067	1·72	1·76–1·68	**<0·001**
BMI/age percentile	54	28	56	77–31	56	26	60	77–34	51	29	51	74–26	0·122
Body weight status*													
* *Underweight													0·718
* n*	3				1				2				
* *%			0·9 %				0·6 %				1·3 %		
* *Normal weight													
* n*	258				139				119				
* *%			79·4 %				80·8 %				77·8 %		
* *Overweight													
* n*	56				29				27				
* *%			17·2 %				16·9 %				17·6 %		
* *Obese													
* n*	8				3				5				
* *%			2·5 %				1·7 %				3·3 %		
Waist circumference (cm)	71·7	8·7	70	75·3–66	70	7·83	69	73–65	73·6	9·25	72	78–67	**<0·001**
Hip circumference (cm)	93·9	7·73	93	98·8–89	94·7	7·93	93·5	99–90	92·9	7·4	92	97–88	**0·017**
Systolic BP (mmHg)	116	12	116	124–109	114	12	114	120–107	120	12	119	125–112	**<0·001**
Diastolic BP (mmHg)	70	8	70	75–66	70	8	70	76–66	70	9	70	75–65	0·361

BP, blood pressure; M, mean; MD, median; IQR, interquartile range.

* Presented as absolute (*n*) and relative frequency (%).

*P* values for gender differences computed using the Chi-square test for body weight status; Mann–Whitney’s test was used for all other comparisons; statistically significant differences (*P* < 0·05) are presented in bold.

**Table 2:** Dietary characteristics of the sample and according to gender

	Total		Female		Male		*P* value
	*n*	%	*n*	%	*n*	%	
Meals/d (*n* 325)							
Breakfast	282	86·8	143	83·1	139	90·8	**0·041**
Mid-morning	232	71·4	134	77·9	98	64·1	**0·006**
Lunch	325	100·0	172	100·0	153	100·0	–
Afternoon	296	91·1	160	93·0	136	88·9	0·192
Dinner	321	98·8	169	98·3	152	99·3	0·373
After dinner meal	131	40·3	55	32·0	76	49·7	**0·001**
Number of meals/d*							
MD	5·0		5·0		5·0		0·275
IQR	6–4		5–4		6–4		
Number of intermediate meals/d*							
MD	2·0		2·0		2·0		**0·005**
IQR	2–1		2–1		2–1		
The five most consumed foods at							
Breakfast (*n* 282)							
Bread	212	75·2	105	73·4	107	77·0	0·490
Milk	211	74·8	96	67·1	115	82·7	**0·003**
Cereals	193	68·4	93	65·0	100	71·9	0·212
Butter, margarine	159	56·4	81	56·6	78	56·1	0·929
Fruit	153	54·3	87	60·8	66	47·5	**0·024**
Mid-morning (*n* 232)							
Bread	164	70·7	94	70·1	70	71·4	0·833
Cakes, cookies	129	55·6	77	57·5	52	53·1	0·505
Varied pastry	104	44·8	65	48·5	39	39·8	0·189
Charcuterie	104	44·8	62	46·3	42	42·9	0·606
Butter, margarine	99	42·7	55	41·0	44	44·9	0·558
Afternoon (*n* 296)							
Bread	241	81·4	128	80·0	113	83·1	0·463
Fruit	176	59·5	105	65·6	71	52·2	**0·019**
Butter, margarine	165	55·7	89	55·6	76	55·9	0·965
Cakes, cookies	162	54·7	89	55·6	73	53·7	0·737
Charcuterie	157	53·0	90	56·3	67	49·3	0·230
Food consumed at							
Lunch (*n* 325)							
Vegetable soup (*n* 325)	159	48·9	93	54·1	66	43·1	**0·049**
Salad (*n* 297)	234	78·5	131	81·9	103	74·6	0·129
Cooked vegetables (*n* 297)	182	61·1	100	68·8	72	52·2	**0·003**
Pulses (*n* 297)	173	58·3	106	66·3	85	61·6	0·403
Sandwich (*n* 325)	100	30·8	52	30·2	48	31·4	0·824
Dessert (*n* 325)	196	60·3	95	55·2	101	66·0	**0·047**
Fruit	165	84·2	85	89·5	80	79·2	**0·049**
Sweet	31	15·8	10	10·5	21	20·8	**0·049**
Beverage (*n* 325)	288	88·6	148	86·0	140	91·5	0·122
Water	279	96·9	144	97·3	135	96·4	0·672
Soft drinks	131	45·5	58	39·2	73	52·1	**0·027**
Fruit nectars	125	43·4	56	37·8	69	49·3	0·050
Beer	14	95·1	0	0·0	14	10·0	**<0·001**
Dinner (*n* 321)							
Vegetal soup (*n* 321)	156	48·6	87	51·5	69	45·4	0·276
Salad (*n* 293)	233	79·5	131	84·0	102	74·5	**0·044**
Cooked vegetables (*n* 293)	192	65·5	115	73·7	77	56·2	**0·002**
Pulses (*n* 293)	163	55·6	101	64·7	86	62·8	0·726
Sandwich (*n* 321)	30	9·3	13	7·7	17	11·2	0·283
Dessert (*n* 321)	202	62·9	104	61·5	98	64·5	0·587
Fruit	155	77·5	84	80·8	71	74·0	0·249
Sweet	45	22·5	20	19·2	25	26·0	0·249
Beverage (*n* 321)	278	86·6	143	84·6	135	88·8	0·270
Water	264	95·0	136	95·1	128	94·8	0·912
Soft drinks	116	41·7	51	35·7	65	48·1	**0·035**
Fruit nectars	106	38·1	46	32·2	60	44·4	**0·035**
Beer	9	3·2	1	.07	8	5·9	**0·014**

* Presented as median (MD) and interquartile range (IQR).

*P* values for gender differences computed with Mann–Whitney’s test for number of meals/d and number of intermediate meals/d; The Chi-square test was used in all other comparisons; statistically significant differences (*P* < 0·05) are presented in bold.

Table 3 presents data regarding physical activity. On the overall, when not at school, boys spend more time in sports activities than girls.

**Table 3:** Participants’ sports activities and sedentary hours during the week

	Total		Female		Male		*P* value
	*n*	%	*n*	%	*n*	%	
School sport (*n* 325)							
No	293	90·2	156	90·7	137	89·5	**0·727**
Yes	32	9·8	16	9·3	16	10·5	
Number of weekly hours (*n* 32)							
1 h	8	25·0	6	37·5	2	12·5	**0·034**
2 h	10	31·3	7	43·8	3	18·8	
3 h	5	15·6	0	0·0	5	31·3	
4 h	5	15·6	1	6·3	4	25·0	
+ 5 h	4	12·5	2	12·5	2	12·5	
Non-academic sport (*n* 325)							
No	145	44·6	97	56·4	48	31·4	**0·011**
Yes	180	55·4	75	43·6	105	68·6	
Number of weekly hours (*n* 180)							
1 h	10	5·6	6	8·1	4	3·8	**0·011**
2 h	40	22·3	24	32·4	16	15·2	
3 h	28	15·6	12	16·2	16	15·2	
4 h	25	14·0	5	6·8	20	19·0	
+ 5 h	76	42·5	27	36·5	49	46·7	
Leisure sports activities (*n* 325)							
No	130	40·0	82	47·7	48	31·4	**0·003**
Yes	195	60·0	90	52·3	105	68·6	
Number of weekly hours (*n* 195)							
1 h	58	29·9	32	36·0	26	24·8	**0·011**
2 h	55	28·4	27	30·3	28	26·7	
3 h	40	20·6	17	19·1	23	21·9	
4 h	21	10·8	11	12·4	10	9·5	
+ 5 h	20	10·3	2	2·2	18	17·1	
Total hours in the week watching television/computer/videogames (*n* 325)							
< 3 h	48	14·8	35	20·3	13	8·5	**<0·001**
3 to 6 h	175	53·8	103	59·9	72	47·1	
7 to 9 h	68	20·9	22	12·8	46	30·1	
≥ 10 h	34	10·5	12	7·0	22	14·4	

*P* values for gender differences computed with Chi-square test; statistically significant differences (*P* < 0·05) are presented in bold.

### Mediterranean diet and health behaviours

The distribution of the KIDMED index categories shows a low adherence to MD group in 9·0 % (*n* 29), intermediate adherence in 45·5 % (*n* 148) and high adherence in 45·5 % (*n* 148) of the participants (Table 4). We did not find gender differences in adherence to MD (*P* = 0·306), but some diet and health behaviours were positively associated with MD. Participants that regularly have breakfast, eat vegetable soup at lunch or dinner, and practice sports and leisure physical activities outside school show a higher median score in the KIDMED index (*P* < 0·001) (Table 5).

**Table 4:** Categories of adherence to the Mediterranean Diet of the sample

	Total (*n* 325)		Female (*n* 172)		Male (*n* 153)		*P* value
	*n*	%	*n*	%	*n*	%	
Low adherence	29	8·9	19	11·0	10	6·5	0·306
Intermediate adherence	148	45·5	74	43·0	74	48·4	
High adherence	148	45·5	79	45·9	69	45·1	

*P* value for gender differences computed with the Chi-square test.

**Table 5:** KIDMED index categories in relation to specific health behaviours

	Total (*n* 325)		Low adherence (*n* 29)		Intermediate adherence (*n* 148)		High adherence (*n* 148)		KIDMED score		*P* value
	*n*	%	*n*	%	*n*	%	*n*	%	MD	IQR	
Breakfast											
No	43	13·2	16	55·2	22	14·9	5	3·4	4	6–3	**<0·001**
Yes	282	86·8	13	44·8	126	85·1	143	96·6	8	9–6	
Vegetable soup											
No	131	40·3	22	75·9	72	48·6	37	25·0	6	8–5	**<0·001**
Yes	194	59·7	7	24·1	76	51·4	111	75·0	8	8–6	
Sport outside the school											
No	145	44·6	24	82·8	70	47·3	51	34·5	6	8–4	**<0·001**
Yes	180	55·4	5	17·2	78	52·7	97	65·5	8	9–6	
Leisure physical activity											
No	130	40·0	23	79·3	66	44·6	41	27·7	6	8–5	**<0·001**
Yes	195	60·0	6	20·7	82	55·4	107	72·3	8	8–6	

MD, median; IQR, interquartile range.

*P* values for comparisons in KIDMED score between ‘No’ and ‘Yes’ groups computed with Mann–Whitney’s test; statistically significant differences (*P* < 0·05) are presented in bold.

Further analyses using the KIDMED index score show a positive correlation between this variable and the number of daily meals (*r*_spearman_ = 0·195, *P* < 0·001) and a negative correlation with sedentary hours per week (*r*_spearman_ =-0·175, *P* = 0·002). No correlations between KIDMED index score and anthropometric characteristics or blood pressure were found (*P* > 0·05).

It is noteworthy that participants with a high adherence to MD usually consume a second piece of fruit daily (80·5 %, *n* 99), fresh or cooked vegetables once (59·4 %, *n* 133) or more than once (70·2 %, *n* 99) daily, and consume nuts at least 2 to 3 times a week (73·2 %, *n* 71) (Table 6).

**Table 6:** KIDMED index items according to score categories of adherence to Mediterranean diet

	Total (*n* 325)		Low adherence (*n* 29)		Intermediate adherence (*n* 148)		High adherence (*n* 148)		*P* value
	*n*	%	*n*	%	*n*	%	*n*	%	
KIDMED index items									
Takes a fruit or fruit juice every day	223	68·6	10	4·5	74	33·2	139	62·3	**<0·001**
Has a second fruit every day	123	37·8	0	0·0	24	19·5	99	80·5	**<0·001**
Has fresh or cooked vegetables regularly once a day	224	68·9	7	3·1	84	37·5	133	59·4	**<0·001**
Has fresh or cooked vegetables more than once a day	141	43·4	2	1·4	40	28·4	99	70·2	**<0·001**
Consumes fish regularly (at least 2–3 times/week)	209	64·3	3	1·4	86	41·1	120	57·4	**<0·001**
Goes more than once a week to a fast-food (hamburger) restaurant	49	15·1	7	14·3	31	63·3	11	22·4	**0·002**
Likes pulses and eats them more than once a week	209	64·3	3	1·4	74	35·4	132	63·2	**<0·001**
Consumes pasta or rice almost every day (5 or more times/week)	237	72·9	21	8·9	105	44·3	111	46·8	0·733
Has cereals or grains (bread, etc.) for breakfast	272	83·7	16	5·9	120	44·1	136	50·0	**<0·001**
Consumes nuts regularly (at least 2–3 times/week)	97	29·8	0	0·0	26	26·8	71	73·2	**<0·001**
Uses olive oil at home	308	94·8	24	7·8	138	44·8	146	47·4	**0·001**
Skips breakfast	40	12·3	16	40·0	21	52·5	3	7·5	**<0·001**
Has a dairy product for breakfast (yoghurt, milk, etc.)	269	82·8	14	5·2	123	45·7	132	49·1	**<0·001**
Has commercially baked goods or pastries for breakfast	72	22·2	10	13·9	35	48·6	27	37·5	0·131
Takes two yoghurts and/or some cheese (40 g) daily	130	40·0	3	2·3	50	38·5	77	59·2	**<0·001**
Takes sweets and candy several times every day	53	16·3	9	17·0	30	56·6	14	26·4	**0·003**
KIDMED index score*									
MD	7		3		6		9		–
IQR	8–5		3–1		7–5		10–8		

* Presented as median (MD) and interquartile range (IQR).

*P* values for group differences in KIDMED categories according to specific dietary habits computed Chi-square tests; statistically significant differences (*P* < 0·05) are presented in bold.

**Fig. 1:**
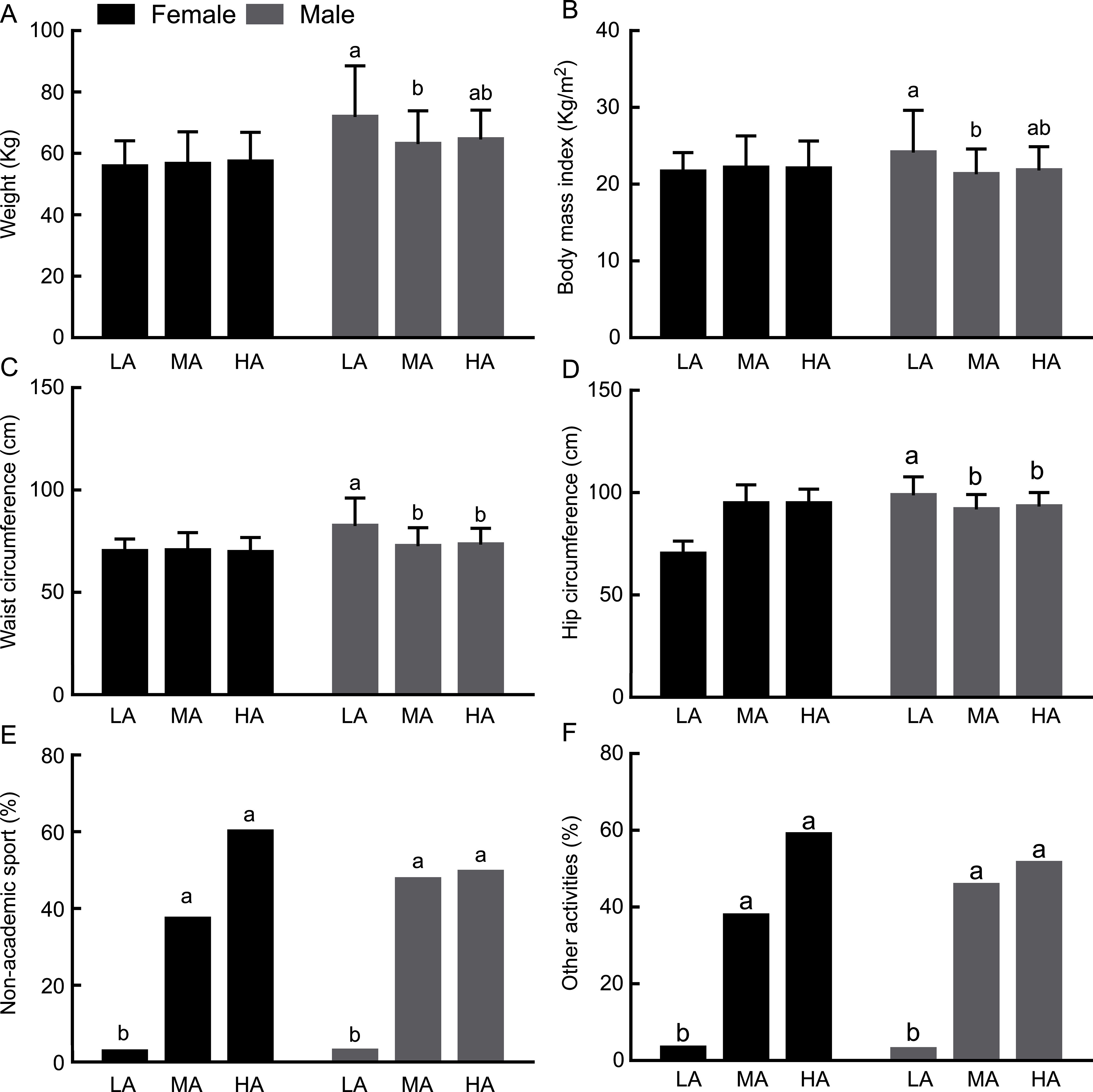
Effect of different levels of adherence to the MD on anthropometric measures and health behaviours (non-academic sports and other physical activities) in boys and girls. The values plotted in bars represent Mean ± sd for the graphic with anthropometric data and percentages for the graphic with physical activities. Different letters on the bars indicate significant differences (one-way ANOVA, post hoc SNK *P* < 0·05) between the Low (L), Medium (M) and high (H) adherence groups analysed in each gender independently

No statistically differences between gender and adherence to the MD were found when the information is considered altogether (Table 1). However, due to the thoroughly investigated metabolic differences between genders, data were split, and an ANOVA test was performed in each gender separately by level of MD Adherence (Fig. 1), which clearly reveals significant differences in the effect of MD adherence in the analysed parameters in each gender. Males with low adherence to MD had significantly higher waist and hip circumference (Fig. 1(c) and (d)). Anthropometric variables and MD were not associated in girls (*P* > 0·05), but girls with high adherence to the MD showed significantly higher healthy behaviours, such as sports outside school and leisure activities (Fig. 1(e) and (f)). In both genders, higher adherence to MD (intermediate and high) is associated with higher adherence to sports outside school and leisure activities (Fig. 1(e) and (f)).

**Fig. 2:**
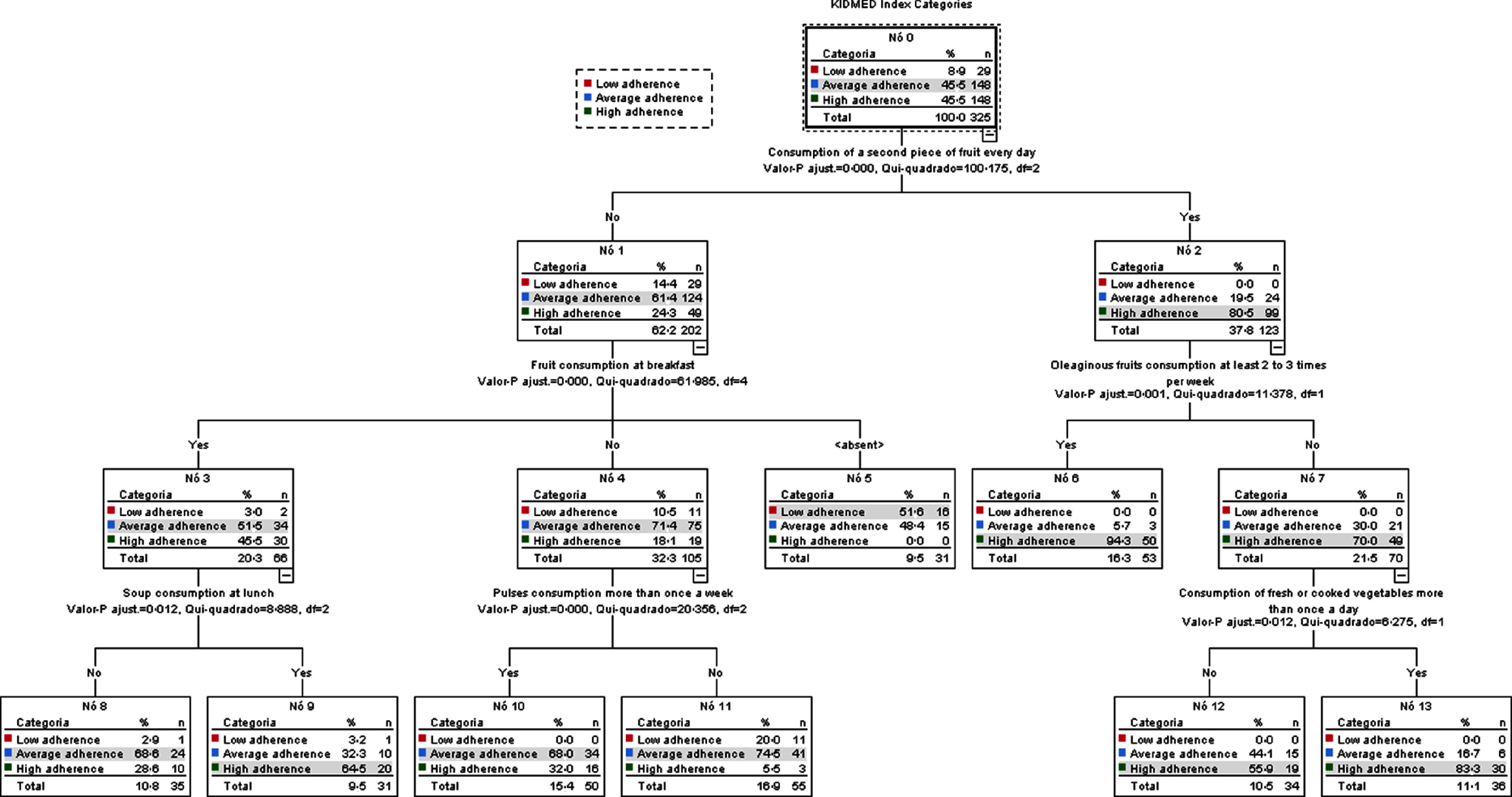
Decision tree obtained through CHAID method to predict which health behaviours contribute to better adherence to the MD in secondary school students. Statistical significance is represented in each tree node, when the tree ramification stops no significant differences are observed within the group. Each node is divided into a group with a significantly higher presence of the prementioned characteristic (e.g. consumption of a second piece of fruit every day) referred as ‘Yes’ or significantly lower presence of individuals with the same characteristic referred as ‘No’. When the tree does not grow from a terminal or a characteristic is not mentioned, means that there are no statistical differences among the analysed categories of KIDMED. Node 5 <absent> represent the individuals that eat a second piece of fruit daily but do not take breakfast

To further analyse the association between adherence to MD and health behaviours, all of the variables related to health behaviours (dietary habits, sleep and oral hygiene, physical activity, socio-demographic data, anthropometric measures and blood pressure) were used in a decision tree analysis through the CHAID method (Fig. 2). This model translated 72 % of correct ratings, which reveals predictive robustness. Considering the categories of the KIDMED index (dependent variable), dietary habits were the only factors that discriminate the categories of adherence to the MD (Fig. 2). The CHAID nodes showed that the daily intake of a second piece of fruit (node 2) is more prevalent in participants with a high adherence to MD (80·5 %, *n* 99). All terminal nodes (6, 12 and 13) derived from node 2 showed a higher prevalence of individuals with high adherence to the MD. The intake of nuts at least 2 to 3 times/week (node 6) and the intake of fresh or cooked vegetables more than once a day (node 13) showed a prevalence of 94·3 % (*n* 50) and 88·3 % (*n* 30), respectively, in participants in high adherence to MD. In the absence of the daily intake of a second piece of fruit, only participants who simultaneously consume fresh fruit for breakfast and vegetable soup for lunch (64·5 %, *n* 20) show high adherence to MD (node 9). In the remaining terminal nodes, participants who do not consume a second piece of fruit daily, despite other intakes, show intermediate adherence to the MD (Fig. 2). The terminal nodes obtained by the decision tree analysis through CHAID are summarised in Table 7.

**Table 7:** Decision rules for the prediction of high adherence to MD and KIDMED index average score

Node	Independent variables						Probability of high adherence to MD (%)	KIDMED index average score
	A	B	C	D	E	F		
6	**Yes**	.	**Yes**	.	.	.	**94·3**	9·53
13	**Yes**	.	No	.	.	No	**83·3**	8·81
9	No	**Yes**	.	**Yes**	.	.	**64·5**	7·58
12	**Yes**	.	No	.	.	No	**55·9**	7·24
10	No	No	.	.	Yes	.	32·0	6·82
8	No	Yes	.	No	.	.	28·6	6·29
11	No	No	.	.	No	.	5·5	4·75

A – consumption of the second piece of fruit every day; B – fruit consumption at breakfast; C – oleaginous fruits consumption at least 2 to 3 times/week; D – soup consumption at lunch; E – pulses consumption more than once a week; F – consumption of fresh or cooked vegetables more than once a day.

## Discussion

Our data show a lower prevalence for overweight (19·7 %) and obesity (2·5 %) than the data reported in the Portuguese national inquiry on food and physical activity, IAN AF 2015–2016, which suggests a national prevalence in adolescents of 23·6 % for overweight and 8·7 % for obesity^(32)^. On the overall, our sample showed an intermediate and high adherence to the MD, a characteristic that is associated in the literature with better nutritional adequacy^(11)^, lower rates of overweight, obesity^(29)^ and lower waist circumference in adolescents^(30,31)^. Our data and the cross-sectional nature of this study do not allow the analysis of cause and effect, but our results add to the body of evidence suggesting a positive statistical association between the MD and health.

Some of the consequences of obesity are gendered, with some evidence that the risk of mortality is higher in adult males who were obese during their adolescence^(47)^. Male adolescents with low adherence to MD had significantly higher waist and hip circumference (*P* < 0·001). As waist circumference provides a simple and practical anthropometric measure to assess central adiposity^(48)^, these data suggest that, in our sample, heavier males have a higher abdominal adiposity and may also suggest that low adherence to a healthy diet can lead to an early onset of overweight and increase the cardiometabolic risk in male adolescents^(49)^. Voltas *et al.* ^(50)^ have found no relationship between anthropometric data and MD adherence during adolescence and state that this association may be reported later in life due to the low adherence to MD that may lead to negative anthropometric effects in the long term, such as higher BMI^(50)^. On the other hand, the MediLIFE Index study suggests that a better adherence to a Mediterranean lifestyle was associated with lower likelihood of being overweight, obese or having abdominal obesity, particularly in those with the highest adherence^(51)^.

Physical activity must also be considered in interpreting these data. In our study, participants with higher adherence to MD (intermediate and high) were more active and these results are in accordance with other studies carried out in southern Italy and Spain, which found that greater adherence to MD was associated with a healthier lifestyle and a higher level of physical activity in adolescents^(52,53)^.

Our results reinforce the role of adequate nutrition and regular physical activity for preventing obesity and shaping the health-related quality of life in adolescents, especially when most recently reviewed studies conducted in southern European countries report that approximately half the children and adolescents show a low adherence to MD^(54)^.

Our study showed low adherence to MD in 9·0 % of individuals, higher than the value found by Adelantado-Renau *et al.* in Spanish adolescents (5·2 %) and, lower than the prevalence found in a study conducted with Sevillian adolescents (16·8 %)^(55)^.

The use of the CHAID method allowed us to find that adherence to MD is more associated with dietary behaviours than all other health behaviours. Our results show that a daily intake of at least two pieces of fruit is the best predictor of ‘high adherence’ to the MD. When a daily second piece of fruit is not consumed, ‘high adherence’ is simultaneously associated with eating fruit at breakfast and vegetable soup at lunch.

Regarding the consumption of fruit, 31·4 % of our sample do not consume fruit daily. This rate is higher than the one found in the HBSC 2018 study, which reports a prevalence of 11·5 % for rarely or never consuming fruit^(56)^, but lower than the prevalence of 78 % for low consumption of fruit and vegetables by Portuguese adolescents, reported in the IAN-AF^(32)^.

The daily consumption of a second piece of fruit, the variable with the highest differentiating power in our CHAID analysis, was reported by 37·8 % of our sample (*n* 123). This prevalence was lower than the one reported by Rito *et al.* (47·9 %) for the same variable, in another recent study also developed in Portugal^(57)^.

Our study shows that 54·3 % (*n* 153) of the individuals eat fruit at breakfast and 48·9 % (*n* 159) eat vegetable soup at lunch. Fruit consumption is one of three elements, together with consumption of cereals and dairy products, that constitute a healthy breakfast^(58)^ which is associated with higher adherence to the MD^(59)^. Vegetables have a strong presence in MD and vegetable soup, a tradition in Portuguese food, has an important contribution to the daily intake of vegetables^(60)^, allowing to achieve the WHO recommendation for daily consumption of 400 g of fruit and vegetables to help prevent chronic disease and nutritional deficiencies^(12)^.

Our results are in accordance with the ones from other studies, which suggest that the promotion of healthy eating habits among adolescents should be a priority. In a study by Chacón-Cuberos *et al.* ^(52)^, adolescents who followed healthy eating habits showed academic benefits, such as better organisational habits, critical thinking, effort and study habits. Boing *et al.* ^(61)^ highlight that school environment interventions may be effective for promoting healthy behaviours and reinforce the importance of the school context on adolescent health. School can facilitate the engagement in healthy behaviour by offering and stimulating opportunities to be healthy (e.g. offer healthy options in the cafeteria, etc) and by promoting barriers to unsafe/unhealthy behaviour (e.g. not selling energy-dense foods in the cafeteria)^(61,62)^. Aarestrup AK *et al.* ^(63)^ found that schools that promote barriers to unsafe/unhealthy behaviours, while having teachers and students who value that policy, show higher rates of programme implementation and acceptance for change. In Portugal, the results of the Eat Mediterranean programme showed a better adherence to the MD after an intervention of educational sessions promoting MD, which proved successful in changing dietary patterns among adolescents. This programme showed an increase in the number of individuals who eat a second piece of fruit every day, as well as an increase in intake of fresh or cooked vegetables and oleaginous fruits^(57)^.

### Strengths and limitations of this study

Our aim was to identify behaviours associated with the MD through a decision tree model, in a representative sample of Algarve adolescents. We consider the strength of this work to be the use of the CHAID method and our results suggest that CHAID can be used in risk analysis and target segmentation for the pre-detection and management of low adherence to MD. According to Song and Lu^(35)^ ‘the decision tree method is a powerful statistical tool for classification, prediction, interpretation and data manipulation that has several potential applications in health research’. The use of decision tree models based on machine learning techniques is a powerful and robust statistically tool for big data analysis, and to support decision-making in the health and human nutrition field.

The main limitations of our study are related with the self-report nature of dietary behaviours and with the limited dietary data collected besides the KIDMED index. Our methods for assessing dietary data do not allow for a proper food frequency or quantitative analysis, and we did not collect information regarding other variables widely known to determine food choice in adolescents, such as parental and peer social support, or other home environment variables.

Unfortunately, we also did not achieve sample size representativity. Our calculations for sample size suggested a minimum of 454 participants and 545 students were invited to be a part of the study. We collected written authorisations from legal guardians of the potential participants in a number far exceeding the minimum sample size. Nevertheless, on the day that data collection was scheduled, a significant number of adolescents, despite having written authorisation from their guardian and having previously agreed to be a part of the study, declined the procedure needed to collect anthropometric data. An analysis (not included in this paper) on the socio-demographic characteristics of the students that declined anthropometric assessment, showed that there were no statistically significant differences between these students and those in the final sample. As per our study protocol, students that did not complete the anthropometric assessment were excluded from the final sample, which totalled 325 participants. This can limit the generalisation of our data, but the non-parametric statistical decision tree approach in this paper can safely be used with our final sample size and variables. Furthermore, the similarities in socio-demographic profile between our final sample and both the non-participants and the overall adolescent population in the Algarve (based on census data from the National Statistics Institute, not show, as it is not within the scope of this paper) suggest that, despite the limitation, our data can contribute to an overall assessment of this region’s adolescents.

## Conclusions

The consumption of nuts and fresh or cooked vegetables was associated with a high adherence to the MD. Through the decision tree methodology, the daily consumption of two portions of fruit and vegetables proved to be the best determinant for high adherence to the MD.

Although adherence to a MD can also be influenced by factors such as parental and peer social support, or by the overall healthfulness of home food environment, schools can provide the framework to a healthy, Mediterranean way of eating, if adequate interventions can be put in place.

The use of the CHAID multivariate tree analysis technique, when accompanied by other statistical analyses, is a promising tool in nutrition-related studies.

The Algarve is a region with traditionally Mediterranean eating habits, and our results allowed us to identify specific habits of MD adherence that should be encouraged in adolescents. Future interventions can be tailored considering the promotion of the daily consumption of two portions of fruit, and vegetables, in school’s settings, to promote a higher adherence to MD and contribute to improving the health and quality of life of adolescents.
